# Aerobic capacity and disease activity in children, adolescents and young adults with juvenile idiopathic arthritis (JIA)

**DOI:** 10.1186/1546-0096-10-27

**Published:** 2012-08-27

**Authors:** Philomien A van Pelt, Tim Takken, Marco van Brussel, Inge de Witte, Aike A Kruize, Nico M Wulffraat

**Affiliations:** 1 Department of Paediatric Immunology, Wilhelmina Children’s Hospital, University Medical Center Utrecht, Utrecht, Netherlands; 2 Department of Rheumatology, Erasmus Medical Center, University Medical Center Rotterdam, Rotterdam, The Netherlands; 3 Child Development & Exercise Center, Wilhelmina Children’s Hospital UMC Utrecht, Utrecht, The Netherlands; 4 Department of Paediatric Immunology, Wilhelmina Children’s Hospital, University Medical Centre Utrecht, Utrecht, Netherlands; 5 Department of Rheumatology & Clinical Immunology, University Medical Center Utrecht, Utrecht, The Netherlands

**Keywords:** Juvenile idiopathic arthritis, Aerobic capacity, Disease activity, Remission, Transition, Adolescents

## Abstract

**Background:**

As patients with juvenile idiopathic arthritis (JIA) progress into adulthood, long-term outcome is determined by disease activity, physical and psychosocial development. Decreased aerobic capacity may play a critical role in health-related outcomes in JIA, since it has been linked with cardiovascular morbidity and mortality in late adulthood. The objectives of the current study are to examine the aerobic capacity and its relation to parameters of disease activity in children, adolescents and young adults with JIA.

**Methods:**

Sixty-three patients with JIA (aged 10–27 years) were cross sectional studied regarding their aerobic capacity and correlations were made to demographic, disease-related variables, and medication utilization. in a cross-sectional study group of 63 patients of all subtypes. Patients were divided in three age groups, 10–13 years; 14–17 years and 18–27 years.

**Results:**

Reduced aerobic capacity is found in clinical remission as well as active disease in all subtypes and all age groups. Aerobic capacity is more impaired in active disease shown by DAS 28, JADAS 27, ESR and serum thrombocyte counts. Lower haemoglobin has a negative impact. Long-term used medication including methotrexate and corticosteroids didn’t influence outcome. There is no association with current sports participation.

**Conclusion:**

Reduced aerobic capacity is present in children and adolescents with JIA, both in active disease and in patients with remission. Measures of aerobic capacity may serve as important outcome measure in JIA.

## Background

Although Juvenile Idiopathic Arthritis (JIA) is generally thought to have a favourable outcome [1], in reality its course may be less propitious. At adult age, persistent disease activity and functional impairment have been reported in more than half of the patients [2-6]. Moreover, disease related chronic inflammation may cause protein loss and fat accumulation [7], increasing the risk for obesity and cardiovascular disease in this population[8]. Systemic inflammation, immune dysfunction and its treatment [9-12], might also contribute to the increased mortality rate in JIA [10,11].

In addition to these disease-related factors [5], factors influencing morbidity in the general population must also be considered as they are likely to have heightened effect on patients with JIA. For example, low aerobic capacity, a marker of physical fitness, may play a critical role in health-related outcomes in JIA since it has been linked with an increased risk of developing chronic diseases including cardiovascular diseases, diabetes, cancer, hypertension, obesity, depression and osteoporosis [13], as well as an increased risk of mortality in adults [14].

Physical fitness is a multidimensional concept defined as a set of attributes that people possess or achieve that relate to the ability to perform physical activity [15]. It is comprised of skill-related (e.g. agility, coordination), health-related (e.g. cardiovascular capacity), and physiological components [16]. In adults, the level of physical fitness is a powerful predictor of mortality in both healthy and disease states [17,18]. The gold standard for assessing physical fitness is the aerobic capacity, commonly defined as the maximal or peak volume of oxygen uptake (VO_2peak_) during an incremental exercise test to exhaustion. VO_2peak_ is expressed either as an absolute value in liters of oxygen per minute (l/min) or as a relative value in milliliters of oxygen per kilogram of bodyweight per minute (ml/kg/min). The relative value is frequently used to compare the fitness levels of patients with a chronic disease including a wide range of ages, body sizes and disease severity.

Aerobic capacity has been shown to be impaired in both younger children and adolescents with JIA [19-21]. These impairments may be associated with a combination of the condition-related pathophysiology itself, its treatment (e.g. medications), hypoactivity and subsequent deconditioning [22]. Other potential contributing factors to reduced aerobic capacity in JIA may include anemia, reduced cardiac output, and muscular dysfunction [20].

Patients with JIA may suffer from normocytic hypochromic anaemia, which implies lower oxygen transport capacity of the blood, and hence, impaired oxygen transport to the muscles. A recent study of children with JIA revealed no significant differences in cardiac output during exercise between the JIA group and healthy controls [23]. Muscle atrophy, however, is a common finding in patients with JIA that may be linked to inactivity, medication utilization (including glucocorticoids) and disease activity [24].

Although its usage is generally related to disease activity and severity, medication itself can indirectly affect aerobic capacity in patients with JIA through its impact on body composition. For example, pharmacological doses of glucocorticoids are associated with protein loss, bone loss and insulin resistance in both hepatic and peripheral tissues, leading to impaired carbohydrate metabolism [18,25].An inverse correlation was found between duration of MTX treatment and respiratory muscle function in children with systemic and polyarticular JIA, possibly explained by a more severe disease course being associated with long-term MTX treatment [26] In contrast to observed findings in adult rheumatoid arthritis (RA) patients [27], in children with JIA, impairment of lung function by MTX is rare[28,29]. Effects of anti-TNF therapy on aerobic capacity are not known yet.

To our knowledge, data on aerobic capacity in relation to the disease activity in patients with JIA are scarce [30]. The aims of our current study are therefore to examine the aerobic capacity and its relation to parameters of disease activity in children, adolescents and young adults with JIA.

## Patients and methods

All consecutive patients between 2005 and 2008 with JIA (as classified by the International League Against Rheumatism criteria, ILAR[31] between the ages of 10 to 27 years were asked to participate in an observational longitudinal cohort study, investigating clinical parameters and aerobic capacity in children, adolescents and young adults with JIA over a 3-year period. A total of 63 patients provided informed consent. The results of baseline assessments are described in the present study. All patients were seen in the out-patient clinics of the paediatric or adult departments of rheumatology & immunology at the University Medical Center (UMC) Utrecht. Patients were divided into three age groups, 10–13 years, 14–17 years and 18–27 years, with the latter patient group recruited from the adult rheumatology department. The middle age group (14–17 years) will be transferred to adult health care over the course of the three-year follow-up study. The Medical-Ethics Committee of the UMC Utrecht approved all study procedures.

Demographic variables, disease characteristics and medication utilization (past and current) were recorded for all patients (Table 1). Functional ability was assessed with the validated Dutch version of the Childhood Health Assessment Questionnaire [32-34]. Patients were asked about which sports and leisure activities were performed and the time consuming per week in a general interview [35]. Physical examination included a general assessment. Standard laboratory values including Erythrocyte Sedimentation Rate (ESR), C - reactive protein (CRP), thrombocytes and haemoglobin were obtained from a blood sample collected at the outpatient clinic. Haemoglobin counts were used to classify anaemia based on international reference values for in children and adolescents [36]. 

**Table 1:** Demographic and disease related variables

**N = number of patients**	**Age 10–13 yrs**	**Age 14–17 yrs**	**Age 18–27 yrs**	**All patients**
	**N = 33**	**N = 18**	**N = 12**	**N = 63**
Boys (N)	13	6	3	22
Girls (N)	20	12	9	41
Subtype JIA N (%)	3 (9.1)	4 (22.2)	1 (8.3)	8 (12.7)
Systemic	3 (9.1)	0 (0)	0(0)	3 (4.8)
Oligo persistent	9 (27.3)	3 (16.7)	3 (25.0)	15 (23.8)
Oligo extended	3 (9.1)	2 (11.1)	2 (16.7 )	7 (11.1)
Polyarticular RF+	14 (42.4)	8 (44.4)	5 (41.7)	27 (42.9)
Polyarticular RF-	0 (0)	1 (5.6)	1 (8.3)	2 (3.2)
Enthesitis related Psoriatic arthritis	1 (3.0)	0 (0)	0 (0)	1 (1.6)
Disease Duration in years (IQR)	5.5 (6.0)	10.5 (6.0)	10.0 (18.0)	8.0 (7.0)
CHAQ (IQR)	1,6 (0.8)	1,4 (0.3)	1,4 (0.6)_	1,5 (0.8)
Sports performance (hours/week, IOR)	2.0 (3.0)	3.0 (8.0)	2.5 (6.0)	2.0 (3.0)
BMI kg/m^2^ (IQR)	17.9 (3.3)	21.3 (4.5)	19.8 (3.9)	18.8 (4.7)
Z-score VO_2peak_ (IQR)	−0.9 (1.5)	−0.8 (1.6)	−0.6 (1.7)	−0.8 (1.3)**
Z-score VO_2peak_/kg (IQR)	−1.6 (1.7)	−1.5 (2.1)	−1.0 (2.1)	−1.5 (1.9)**
ESR mm (IQR)	8.0 (10.0)	9.5 (16.0)	7.0 (14.0)	8.0 (13.0)
CRP mg/l (IQR)	2.0 (5.0)	6.0 (23.0)	6.0 (5.0)	5.5 (6.0)
Haemoglobin mmol/l (IQR)	8.0 (1.0)	7.6 (1.0)	8.7 (1.3)	7.9 (1.3)
Thrombocytes 10.9/l (IQR)	315 (102)	273 (102)	290 (93)	298 (83)
DAS28 (IQR)	1.7 (1.1)	1.8 (1.9)	1.5 (1.6)	1.6 (1.2)
JADAS 27 (IQR)	3.6 (7.1)	3.0 (22.9)	2.6 (17.1)	2.9 (9.0)
VAS patient (IQR)	0.2 (2.3)	0.5 (3.1)	0.5 (.6)	0.5 (2.3)
Number of swollen joints (IQR)	0.0 (2.0)	0.0 (3.0)	0.0 (3.0)	0.0 (4.4)
Number of tender joints (IQR)	0.0 (3.5)	0.0 (4.0)	0.0 (3.8)	0.0 (4.1)
Number of limited joints (IQR)	1.5 (4.0)	2.0 (5.0)	2.5 (5.0)	2.0 (4.0)

All variables are expressed as median values unless otherwise stated.

** p < 0.01 significant difference compared to healthy age and sex related individuals.

### Aerobic capacity

Aerobic capacity (VO_2peak_) was assessed using a graded cardiopulmonary exercise test (CPET) to volitional exhaustion performed on an electronically braked cycle ergometer (Lode examiner, Lode BV, Groningen, the Netherlands). The seat height was adjusted to the patient’s comfort. Patients began cycling at a workload of 0 Watts and this increased by 20 Watts every minute until they were no longer able to continue cycling due to volitional exhaustion, despite strong verbal encouragement. Patients breathed through a mouthpiece, which was connected to a calibrated metabolic cart (Oxycon Pro, Care Fusion, Houten, the Netherlands). Expired gases were collected and analysed for breath-by-breath minute ventilation (Ve), oxygen uptake (VO_2_), carbon dioxide exhalation (VCO_2_) and respiratory exchange ratio (RER; =VCO_2_/VO_2_) using conventional equations. The highest achieved oxygen uptake averaged over a 30-second period was taken as VO_2peak._ Heart rate (HR) was measured continuously via 3-lead ECG. To allow comparison of aerobic capacity with healthy controls, standardized z-scores were determined using: Z-scores *= [(observed VO*_*2peak*_*- predicted VO*_*2peak*_*) / SD]* where predicted VO_2peak_ values were obtained from a database of established values for age- and gender- related Dutch controls [37]. A z-score of zero represents a VO_2_ similar to that of healthy controls, while a score of ±1.5 is a clinical important difference from healthy controls.

### Disease activity

A number of indicators were used to assess disease activity including the total number of swollen joints, number of painful joints, the number of joints with limited range of motion, number of active joints (defined as joints with swelling and/ or pain during motion), the Juvenile Arthritis Disease Activity Score (JADAS 27) [38] and the Disease Activity Index 28 (DAS28)[39]. All of the examinations were performed by an experienced (paediatric) rheumatologist. Furthermore, a global assessment was performed by the patient (well being; VAS patient 0-100 mm) as well by a paediatric rheumatologist (disease activity; PGA 0-100 mm). Given that patients were recruited from both the paediatric and adult clinics, disease activity scores commonly used in both the paediatric and adult departments were computed. In adult Rheumatoid Arthritis (RA) patients, the DAS28 is a commonly used and validated tool to measure disease activity [39]. This is a compound score of the number of a total of swollen joints (out of 28), the number of a total of painful joints (out of 28), assessment of patient’s general health and the erythrocyte sedimentation rate (ESR). Three compound disease activity scores were recently introduced in paediatric rheumatology: Juvenile Arthritis Disease Activity Score (JADAS) 10, JADAS 27 and JADAS 71[38]. We utilised the JADAS 27 compound score because of its feasibility. Moreover, statistical performance of JADAS 27 is comparable to JADAS 71, while the construct validity of JADAS 10 is poor compared to JADAS 71 and JADAS 27 [38]. The JADAS 27 may present an advantage over the DAS 28 because it includes measures for the cervical spine, hips and ankles, joints which are often affected in (adult) patients with JIA.

### Medication use

Past and current medication utilization was obtained from a review of the patient’s medical chart.

### *Statistical analysis*

All statistical analyses were performed using Statistical Package for Social Science (SPSS) version 15.0 for windows (SPSS Inc., Chicago, IL) version 15.0 for Windows. Because patient data were non normally distributed non-parametric tests were used to analyse the demographic variables and compare the results of the cardiopulmonary exercise tests (Mann–Whitney and Kruskal-Wallis where appropriate). Spearman’s Correlation test was used for determining the correlations between VO_2peak_ and disease activity markers and standard laboratory values. The level of statistical significance was set at P < 0.05.

## Results

### Demographics

Demographic and disease related characteristics are presented in Table 1. In total, 22 boys and 41 girls participated in the study. The longest disease durations were seen in the oldest age group. No significant differences were noted between age groups with respect to disease duration, body mass index (BMI), disease activity markers as measured by number of swollen and tender joints, DAS28, JADAS 27 and laboratory values.

### Aerobic capacity

**Figure 1:**
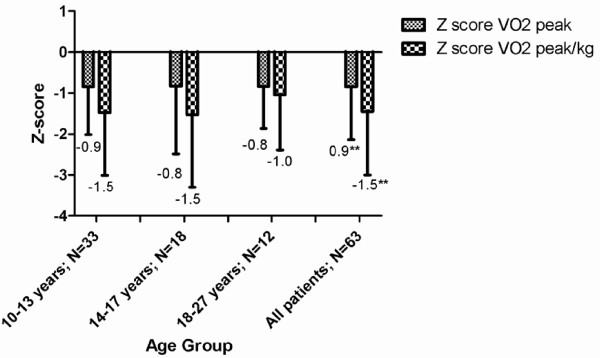
**Aerobic capacity (expressed as median Z-score of VO**_**2peak**_**and VO**_**2peak**_**/kg) in relation to age in adolescents and young adults with JIA.** Z scores are compared to age and sex related healthy controls (p-values <0.01). Abbreviations: Z-score_:_ standard deviation of the individual patient compared with values from age- and sex- related historical Dutch controls(37); VO_2peak abs_: highest oxygen uptake in liters of oxygen per minute (l/min) during maximum exercise test in l/min; VO_2peak_/kg_l_ : highest oxygen uptake in milliliters of oxygen per kilogram of bodyweight per minute (ml/min/kg) during maximum exercise.

The median peak heart rate was 187 beats per minute (Interquartile range [IQR] 17), indicating an adequate level of maximum achieved exercise. The median aerobic capacity in all patients with JIA was considerably lower compared to healthy peers, with a median z-score of −0.8 (p < 0.01) for absolute VO_2peak_ (L/min) and −1.5 (p < 0.01) for relative VO_2peak_/kg (ml/kg/min) in all patients. VO_2peak_ did not differ significantly by gender, disease duration or JIA sub-type; therefore all patients were merged for further analysis. There were no statistical significant differences in the z-scores for absolute and relative VO_2peak_ between the different age groups (Figure 1).

### Aerobic capacity and disease activity

Spearman’s correlation coefficient revealed a significant negative correlation between the number of swollen joints and absolute VO_2peak_ z-score, but not with relative VO_2peak_ z-score (Table 2). No significant correlations were seen between VO_2peak_ values and the number of painful joints. Both disease severity compound scores (DAS28 and JADAS 27) showed significant moderately strong negative associations with all VO_2peak_ values (Table 2).

**Table 2:** Median values (IQR) of Disease activity markers and Spearman’s Correlation Coefficient values for Aerobic Capacity, expressed by absolute and relative VO2 peak

	**Median (IQR)**	**Correlation**	**Correlation**
		**Z-score VO**_**2peak**_	**Z–score VO**_**2peak**_**/kg**
		**(p -value)**	**(p -value)**
Number of swollen joints in lower extremities	0.0 (0)	−0.3 (**)	−0.1
number of swollen joints in upper and lower extremities	0.0 (2)	−0.3 (**)	−0.1
Number of painful joints lower extremities	0.0 (2)	−0.1	−0.1
number of painful joints in upper and lower extremities	0.0 (4.0)	−0.2	−0.1
number of limited joints in upper and lower extremities	2.0 (4.0))	−0.3 (*)	−0.3 (*)
Active joint count	0.0 (0)	−0.3 (**)	−0.2
VAS patient	0.5 (2.6)	−0.3 (*)	−0.3 (*)
DAS28	1.6 (1.2)	−0.4 (**)	−0.4 (**)
JADAS 27	2.9 (9.0)	−0.3(**)	−0.4(**)

level of significance:* indicates significance < 0.05; ** indicates significance <0.01.

**Figure 2:**
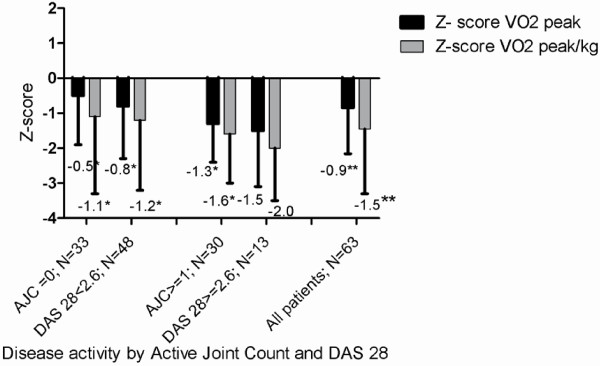
**Aerobic capacity (expressed as Z-score of VO**_**2peak**_**and VO**_**2peak**_**/kg) in relation to disease activity in adolescents and young adults with JIA in remission and with active disease.** Z scores are compared to age and sex matched healthy controls (* p-values <0.05; **p-values <0.01). Abbreviations: Z-score_:_ standard deviation of the individual patient compared with values from age- and sex- related historical Dutch controls(37); VO_2peak abs_: highest oxygen uptake in liters of oxygen per minute (l/min) during maximum exercise test in l/min; VO_2peak_/kg_l_ : highest oxygen uptake in milliliters of oxygen per kilogram of bodyweight per minute (ml/min/kg) during maximum exercise; AJC: Active Joint Count; DAS28: Disease Activity Score of 28 joints.

A substantial proportion of the patients presented with low disease activity as reflected by low median numbers of swollen and painful joints (Table 2). The patients were divided into 2 groups based on disease activity status, these included patients in remission or inactive disease (defined by Wallace et al. [40], active joint count zero; DAS28 ≤2.6) and a patient group with disease activity (active joint count one and higher; DAS28 > 2.6) [40,41]. Between these patient groups, significant differences as measured by active joint count and DAS28 score were seen (Figure 2). 

Significant negative correlations were observed between laboratory parameters reflecting disease activity (ESR, thrombocytes) and the aerobic capacity (Table 3). Conversely, no relationship was seen between C-reactive protein, an acute phase protein and VO_2peak_ (Table 3). We found a positive significant correlation between serum haemoglobin and the relative VO_2peak_/kg in boys as well in the total patient group. In girls, a positive significant correlation between serum haemoglobin and the absolute VO_2peak_ (p < 0.05, table 3) was observed. Lower VO_2peak_ and VO_2peak_/kg values were noted in patients with anaemia compared to those who were not anaemic (p < 0.05).

**Table 3:** Median (IQR) Standard Laboratory Values and Spearman’s Correlation Coefficients for Aerobic Capacity (expressed by absolute and relative VO2 peak) (level of significance:* indicates significance < 0.05; ** indicates significance <0.01)

	**Median (IQR)**	**Correlation with**	**Correlation**
		**Z-score VO**_**2peak**_**(p -value)**	**Z-score VO**_**2peak**_**/kg (p -value)**
ESR (mm)	8.0 (13)	−0.3 (*)	−0.5 (**)
CRP (mg/l)	5.5 (6)	−0.2	−0.2
Thrombocytes (10.9/l)	302 (110)	−0.4 (**)	−0.5(**)
Haemoglobin (mmol/l) boys	8.1 (1.2)	+0.0	+0.5 (*)
Haemoglobin (mmol/l) girls	7.8 (1.1)	+0.3 (*)	+0.2

Legend: Haemoglobin boys (ref: age group 10-12 yrs: 6.6-.8.4 millimol per liter age 12–18 yrs 6.9-9.1 millimol per liter; age >18 yrs: 8.6-10.5 millimol per liter); Hemoglobin girls (ref: age 10–12 yrs 6.6-8.4 millimol per liter ; age 12–18 yrs 6.8-8.3 millimol per liter ; age >18 yrs:7.5-9.5 millimol per liter).

### Aerobic Capacity and Use of Disease Modifying Anti-Rheumatic Drugs (DMARD)

Thirteen of our patients were not using any DMARD at the time of the testing, 38 patients were using one DMARD, 11 patients were using a combination of two DMARD’s and only 1 patient was on a combination therapy of three DMARD’s. Non-parametric tests revealed no significant differences in aerobic capacity between these patients. No significant correlations were found between current use of DMARD and absolute and relative VO_2peak_ values. When looking at historical DMARD use, only use of anti-Tumor Necrosing Factor biologicals in 10 patients was significantly associated with lower absolute and relative VO_2peak_ values (p < 0.05). Duration of current and formerly MTX use did not affect aerobic capacity.

A total of 6 patients were currently using corticosteroids at the time of testing, while 10 patients had used them the past and 47 patients never used corticosteroids. Neither current nor former corticosteroid use was significantly related to aerobic capacity.

## Discussion

Although a favorable long-term outcome is seen in about half of the patients with JIA [1], results of the current study highlight a significant impairment in aerobic capacity, with a median z score of −1.5 in all patients (children, adolescents and young adults with JIA. Although disease duration and DMARD use were expected to have a negative influence on aerobic capacity, no significant correlations, with the exception of past biological use were seen between these variables.

Despite the overall low median disease activity in our patient group, representing the general favorable disease outcome, aerobic capacity was still significantly reduced in patients with JIA compared to healthy controls. Significant impairments in aerobic capacity have previously been reported in youth with other chronic diseases including Crohn’ disease and dermatomyositis [42-44]. A significant correlation of disease activity with aerobic capacity was found (Table 2), the strongest correlations were seen for the compound scores DAS28 and JADAS27. When considering separate items, aerobic capacity was significantly related to the number of swollen, limited, and active joints, but not to the number of painful joints. Apparently, joint-pain is a negligible limiting factor in reaching a maximum of exercise levels.

When the study population was divided by disease activity status, (remission or inactive disease [40] versus active disease) significant differences in aerobic capacity were observed (Figure 2). Since a lower aerobic capacity was also noted in patients in remission compared with healthy controls, one might hypothesize that current indicators of disease activity in JIA are not always sufficient to predict impaired aerobic capacity. As low aerobic capacity is linked with an increased risk of developing other chronic diseases including cardiovascular diseases, diabetes, cancer, hypertension, obesity, depression and osteoporosis [13], as well as an increased risk of mortality in adults [14], this current finding is of importance for the quality of life of all patients with JIA.

Significantly negative correlations were observed between ESR, thrombocytes and both absolute and relative aerobic capacity, suggesting that disease activity and systemic inflammation may negatively affect aerobic capacity. Extensive studies on the role of inflammatory cytokines (e.g. TNF-α) in maintaining the balance between muscle protein synthesis and degradation [7] have lead to the concept of *rheumatoid cachexia*, a condition in which muscle protein degradation is favored over synthesis resulting in muscle wasting, impaired muscle strength and a concomitant increase of fat mass. Body mass index was used as an indirect parameter of body composition in the current study, but did not reveal any significant relationship with aerobic capacity. Although it has been described a common complication in JIA [45], we did not find a significant growth retardation (median length 50^th^ percentile; median weight 49^th^ percentile) in our population.

Future studies in patients with JIA might also consider the inclusion of detailed determination of body composition (muscle mass, fat mass and bone mass) next to muscle strength testing.

This is the first study in patients with JIA to show lower aerobic capacity in individuals with lower serum hemoglobin .This correlation may be explained by a lower oxygen transport capacity to the muscles. In chronic conditions like end-stage kidney disease, anemia is thought to at least partly explain the observed reduction in aerobic capacity [46]. Anemia is often considered a reflection of disease activity [47]*,* suggesting that perhaps the observed correlation with aerobic capacity is due to disease activity, rather than anaemia per se; however, this relationship remained significant when the data were corrected for disease activity.

In our study, no evidence for the influence of any DMARD or glucocorticoids intake on aerobic capacity was found, and also duration of MTX treatment was not correlated with VO_2peak._ Although in earlier days MTX treatment was limited to more severe disease course, in our study population, MTX is used in all subtypes of JIA and in almost all patients (61 out of 63 patients). Only past anti-TNF biological therapy appeared to negatively influence aerobic capacity; this may be related to the fact that anti-TNF in the Netherlands is restricted to patients with a poly-articular disease course after failure of treatment with, or untreatable side effects of high dose MTX. This suggests that a more severe disease course, rather than biological therapy, per se, is associated with a lower aerobic capacity. It is important to note that despite our finding of similar disease activity among all JIA subtypes and DMARD utilization, generally patients with a poly-articular and systemic JIA are considered to have a more severe disease course.

In long standing JIA muscle atrophy, decreased muscle strength and lower extremity deformities might serve to limit aerobic capacity. Moreover much like adults with RA, patients with JIA may have low levels of physical activity (hypo activity) [22], highlighting the need for more rigorous training programs in this population to prevent the increased risk of developing chronic diseases like cardiovascular disease and diabetes [13,48,49]. In the present study, approximately 73% of the patients reported participating in sport more than 12 times a year, compared with 68% in general population [35]. In our study, sport participation was not significantly correlated with aerobic capacity (results not shown).

We realize that the current patient group represents a select group of patients that may not be reflective of the general pediatric and young adult rheumatology population. More specifically, since the patients in our study, are at least 10 years of age, the percentage of persistent oligo-articular patients is lower than the proportion typically seen in the pediatric outpatient clinic. Despite this patient selection, it is remarkable that a negative correlation was observed between disease activity and aerobic capacity, which persisted into adulthood. Furthermore it is also important to note that although a number of correlations presented were only moderately strong, they remained significant and were consistent across several markers that reflect disease activity, namely DAS 28, JADAS 27, and laboratory markers like ESR, thrombocytes and hemoglobin.

## Conclusions

Our results demonstrate that children, adolescents and young adults with JIA suffer from reduced aerobic capacity. This impairment in aerobic capacity was seen in patients with active disease as well as those in remission. Further, we report that aerobic capacity is negatively correlated to disease activity, as measured by physical examination as well as several laboratory parameters. Given that poor aerobic capacity has been linked with a number of healthy complications, these findings highlight the need for longstanding follow up into adulthood of patients with JIA, even if disease activity is low or in remission.

## Abbreviations

JIA: Juvenile Idiopathic Arthritis; IQR: interquartile range; CHAQ: Childhood Health Assessment Questionnaire Sports performance; BMI: Body Mass Index; Z-score: standard deviation of the individual patient compared with values from age- and sex- related historical Dutch controls; VO_2peak abs_: highest oxygen uptake in liters of oxygen per minute (l/min) during maximum exercise test in l/min; VO_2peak_/kg: highest oxygen uptake in milliliters of oxygen per kilogram of bodyweight per minute (ml/min/kg) during maximum exercise; ESR: Erythrocyte Sedimentation Rate; CRP: C-Reactive Protein; DAS28: Disease Activity Score of 28 joints; JADAS 27: Juvenile Arthritis Disease Activity Score of 27 joints; VAS patient: Visual Analogue Scale of General Health.

## Competing interests

The authors declare that they have no competing interests.

## Authors’ contributions

NW, PP and AK participated in the design of the study. TT and MB carried out the aerobic capacity studies. PP, NW, AK and MW performed the JIA disease activity analysis and drafted the manuscript. PP and TT performed the statistical analysis. All authors read and approved the final manuscript.

## References

[B1] Flato B Aasland A Vinje O Forre O Outcome and predictive factors in juvenile rheumatoid arthritis and juvenile spondyloarthropathy J Rheumatol 1998 25 2 366 375 9489836

[B2] Fantini F Gerloni V Gattinara M Cimaz R Arnoldi C Lupi E Remission in juvenile chronic arthritis: a cohort study of 683 consecutive cases with a mean 10 year followup J Rheumatol 2003 30 3 579 584 12610820

[B3] Packham JC Hall MA Long-term follow-up of 246 adults with juvenile idiopathic arthritis: functional outcome Rheumatology (Oxford) 2002 41 12 1428 1435 10.1093/rheumatology/41.12.1428 12468825

[B4] Oen K Malleson PN Cabral DA Rosenberg AM Petty RE Cheang M Disease course and outcome of juvenile rheumatoid arthritis in a multicenter cohort J Rheumatol 2002 29 9 1989 1999 12233897

[B5] Solari N Viola S Pistorio A Magni-Manzoni S Vitale R Ruperto N Assessing current outcomes of juvenile idiopathic arthritis: a cross-sectional study in a tertiary center sample Arthritis Rheum 2008 59 11 1571 1579 10.1002/art.24202 18975357

[B6] Minden K Niewerth M Listing J Biedermann T Bollow M Schontube M Long-term outcome in patients with juvenile idiopathic arthritis Arthritis Rheum 2002 46 9 2392 2401 10.1002/art.10444 12355487

[B7] Roubenoff R Roubenoff RA Cannon JG Kehayias JJ Zhuang H wson-Hughes B Rheumatoid cachexia: cytokine-driven hypermetabolism accompanying reduced body cell mass in chronic inflammation J Clin Invest 1994 93 6 2379 2386 10.1172/JCI117244 8200971 PMC294444

[B8] D'Agostino RB Sr Vasan RS Pencina MJ Wolf PA Cobain M Massaro JM General cardiovascular risk profile for use in primary care: the Framingham Heart Study Circulation 2008 117 6 743 753 10.1161/CIRCULATIONAHA.107.699579 18212285

[B9] John H Kitas G Toms T Goodson N Cardiovascular co-morbidity in early rheumatoid arthritis Best Pract Res Clin Rheumatol 2009 23 1 71 82 10.1016/j.berh.2008.11.007 19233047

[B10] Gabriel SE Why do people with rheumatoid arthritis still die prematurely? Ann Rheum Dis 2008 67 3 30 34 10.1136/ard.2008.098038 PMC2830861 19022810

[B11] Gabriel SE Michaud K Epidemiological studies in incidence, prevalence, mortality, and comorbidity of the rheumatic diseases Arthritis Res Ther 2009 11 3 229 10.1186/ar2669 19519924 PMC2714099

[B12] French AR Mason T Nelson AM O'Fallon WM Gabriel SE Increased mortality in adults with a history of juvenile rheumatoid arthritis: a population-based study Arthritis Rheum 2001 44 3 523 527 10.1002/1529-0131(200103)44:3<523::AID-ANR99>3.0.CO;2-1 11263765

[B13] Warburton DE Nicol CW Bredin SS Health benefits of physical activity: the evidence CMAJ 2006 174 6 801 809 10.1503/cmaj.051351 16534088 PMC1402378

[B14] Byberg L Melhus H Gedeborg R Sundstrom J Ahlbom A Zethelius B Total mortality after changes in leisure time physical activity in 50 year old men: 35 year follow-up of population based cohort Br J Sports Med 2009 43 7 482 19581403

[B15] Caspersen CJ Powell KE Christenson GM Physical activity, exercise, and physical fitness: definitions and distinctions for health-related research Public Health Rep 1985 100 2 126 131 3920711 PMC1424733

[B16] Anonymous Presidents council on Physical fitness Research Digest 2000

[B17] Blair SN Cheng Y Holder JS Is physical activity or physical fitness more important in defining health benefits? Med Sci Sports Exerc 2001 33 6 Suppl S379 S399 11427763 10.1097/00005768-200106001-00007

[B18] Myers J Prakash M Froelicher V Do D Partington S Atwood JE Exercise capacity and mortality among men referred for exercise testing N Engl J Med 2002 346 11 793 801 10.1056/NEJMoa011858 11893790

[B19] van Brussel M van Lelieveld OT van der OT Engelbert Lelieveld RH Helders PJ Tskken T Aerobic and anaerobic exercise capacity in children with juvenile idiopathic arthritis Arthritis Rheum 2007 57 6 891 897 10.1002/art.22893 17665476

[B20] Takken T Hemel A Van der NJ Helders PJ Aerobic fitness in children with juvenile idiopathic arthritis: a systematic review J Rheumatol 2002 29 12 2643 2647 12465166

[B21] Lelieveld OT van Brussel M Takken T van Weert E van Leeuwen MA Armbrust W Aerobic and anaerobic exercise capacity in adolescents with juvenile idiopathic arthritis Arthritis Rheum 2007 57 6 898 904 10.1002/art.22897 17665473

[B22] Lelieveld OT Armbrust W van Leeuwen MA Duppen N Geertzen JH Sauer PJ Physical activity in adolescents with juvenile idiopathic arthritis Arthritis Rheum 2008 59 10 1379 1384 10.1002/art.24102 18821655

[B23] Houghton KM Potts JE Sheel AW Petty RE McKenzie DC Aerobic and anaerobic capacity in Juvenile Idipatic Arthritis: evaluation of the cardiorespiratory response Internet J Rheumatol 2008 5 2 10.5580/201f

[B24] Lindehammar H Sandstedt P Measurement of quadriceps muscle strength and bulk in juvenile chronic arthritis. A prospective, longitudinal, 2 year survey J Rheumatol 1998 25 11 2240 2248 9818671

[B25] Mauras N Growth hormone therapy in the glucocorticosteroid-dependent child: metabolic and linear growth effects Horm Res 2001 56 Suppl 1 13 18 11786679 10.1159/000048128

[B26] Knook LM de Kleer IM van der Ent CK van der Net JJ Prakken BJ Kuis W Lung function abnormalities and respiratory muscle weakness in children with juvenile chronic arthritis Eur Respir J 1999 14 3 529 533 10.1034/j.1399-3003.1999.14c09.x 10543271

[B27] Khadadah ME Jayakrishnan B Al Gorair S Al Mutairi M Al Maradni N Onadeko B Effect of methotrexate on pulmonary function in patients with rheumatoid arthritis–a prospective study Rheumatol Int 2002 22 5 204 207 10.1007/s00296-002-0227-6 12215867

[B28] Schmeling H Stephan V Burdach S Horneff G Pulmonary function in children with juvenile idiopathic arthritis and effects of methotrexate therapy Z Rheumatol 2002 61 2 168 172 10.1007/s003930200025 12056294

[B29] Camiciottoli G Trapani S Castellani W Ginanni R Ermini M Falcini F Effect on lung function of methotrexate and non-steroid anti-inflammatory drugs in children with juvenile rheumatoid arthritis Rheumatol Int 1998 18 1 11 16 10.1007/s002960050047 9672993

[B30] Metin G Ozturk L Kasapcopur O Apelyan M Arisoy N Cardiopulmonary exercise testing in juvenile idiopathic arthritis J Rheumatol 2004 31 9 1834 1839 15338509

[B31] Petty RE Southwood TR Manners P Baum J Glass DN Goldenberg J International League of Associations for Rheumatology classification of juvenile idiopathic arthritis: second revision, Edmonton, 2001 J Rheumatol 2004 31 2 390 392 14760812

[B32] Singh G Athreya BH Fries JF Goldsmith DP Measurement of health status in children with juvenile rheumatoid arthritis Arthritis Rheum 1994 37 12 1761 1769 10.1002/art.1780371209 7986222

[B33] Ruperto N Ravelli A Pistorio A Malattia C Cavuto S Gado-West L Cross-cultural adaptation and psychometric evaluation of the Childhood Health Assessment Questionnaire (CHAQ) and the Child Health Questionnaire (CHQ) in 32 countries. Review of the general methodology Clin Exp Rheumatol 2001 19 4 Suppl 23 S1 S9 11510308

[B34] Wulffraat N van der Net JJ Ruperto N Kamphuis S Prakken BJ ten Cate R The Dutch version of the Childhood Health Assessment Questionnaire (CHAQ) and the Child Health Questionnaire (CHQ) Clin Exp Rheumatol 2001 19 4 Suppl 23 S111 S115 11510312

[B35] Dool R Elling A Hoekman R *Mulier Instituut iov NOC*NSF* Sporters Monitor 2008; description of actual sportissues 2009 1 124

[B36] Brugnara C Nathan DG, Orkin SH, Ginsburg D Reference values in early childhood Hematology of infancy and childhood 2003 Sixth Saunders, Philadelphia, USA 1835 1864

[B37] Binkhorst RA van Hof MA Saris WHM Maximale inspanning door kinderen; referentiewaarden voor 6–18 jarige meisjes en jongens [Maximal exercise in children; reference values girls and boys, 6–18 year of age] 1992 Nederlandse Hartstichting, Den-Haag

[B38] Consolaro A Ruperto N Bazso A Pistorio A Magni-Manzoni S Filocamo G Development and validation of a composite disease activity score for juvenile idiopathic arthritis Arthritis Rheum 2009 61 5 658 666 10.1002/art.24516 19405003

[B39] Prevoo ML Hof MA Kuper HH van Leeuwen MA van de Putte LB van Riel PL Modified disease activity scores that include twenty-eight-joint counts. Development and validation in a prospective longitudinal study of patients with rheumatoid arthritis Arthritis Rheum 1995 38 1 44 48 10.1002/art.1780380107 7818570

[B40] Wallace CA Ruperto N Giannini E Preliminary criteria for clinical remission for select categories of juvenile idiopathic arthritis J Rheumatol 2004 31 11 2290 2294 15517647

[B41] Fransen J Creemers MC van Riel PL Remission in rheumatoid arthritis: agreement of the disease activity score (DAS28) with the ARA preliminary remission criteria Rheumatology (Oxford) 2004 43 10 1252 1255 10.1093/rheumatology/keh297 15238643

[B42] Takken T Spermon N Helders PJ Prakken AB Van der NJ Aerobic exercise capacity in patients with juvenile dermatomyositis J Rheumatol 2003 30 5 1075 1080 12734909

[B43] Takken T Terlingen HC Helders PJ Pruijs H van der Ent CK Engelbert RH Cardiopulmonary fitness and muscle strength in patients with osteogenesis imperfecta type I J Pediatr 2004 145 6 813 818 10.1016/j.jpeds.2004.08.003 15580207

[B44] Ploeger HE Takken T Wilk B Issenman RM Sears R Suri S Exercise Capacity in Pediatric Patients with Inflammatory Bowel Disease J Pediatr 2011 158 5 814 819 10.1016/j.jpeds.2010.10.020 21146188

[B45] Minden K Adult outcomes of patients with juvenile idiopathic arthritis Horm Res 2009 72 Suppl 1 20 25 19940491 10.1159/000229759

[B46] Konstantinidou E Koukouvou G Kouidi E Deligiannis A Tourkantonis A Exercise training in patients with end-stage renal disease on hemodialysis: comparison of three rehabilitation programs J Rehabil Med 2002 34 1 40 45 10.1080/165019702317242695 11900261

[B47] Harvey AR Pippard MJ Ansell BM Microcytic anaemia in juvenile chronic arthritis Scand J Rheumatol 1987 16 1 53 59 3589590

[B48] Lemmey AB Marcora SM Chester K Wilson S Casanova F Maddison PJ Effects of high-intensity resistance training in patients with rheumatoid arthritis: a randomized controlled trial Arthritis Rheum 2009 61 12 1726 1734 10.1002/art.24891 19950325

[B49] Gualano B Sa Pinto AL Perondi B Leite Prado DM Omori C Ammeida RT Evidence for prescribing exercise as treatment in pediatric rheumatic diseases Rev Autoimmun 2010 9 8 569 573 10.1016/j.autrev.2010.04.001 20388559

